# Identifying patients at high risk of decompensated liver disease through unscheduled care attendance data: a retrospective cohort study

**DOI:** 10.1186/s12876-025-04534-2

**Published:** 2026-01-21

**Authors:** R. Swann, J. Lewsey, D. Jamieson, S. Padmanabhan, J. P. Pell, D. Mackay, R. Dundas, J. M. Friday, T. Q. B. Tran, D. Brown, F. K. Ho, C. Hastie, M. Fleming, C. Geue, A. Stevenson, C. du Toit, A. Fraser, E. H. Forrest

**Affiliations:** 1 NHS Greater Glasgow and Clyde, Glasgow, UK; 2 School of Health & Wellbeing, University of Glasgow, Glasgow, UK; 3 School of Cardiovascular & Metabolic Health, University of Glasgow, Glasgow, UK; 4 Digital Health Validation Lab, Living Laboratory, University of Glasgow, Glasgow, UK

**Keywords:** Cirrhosis, Liver disease, Alcohol related liver disease

## Abstract

**Background:**

Liver cirrhosis is one of the leading causes of mortality and morbidity in those of working age. Mortality from liver disease in the UK has continued to rise over the past decade. A significant proportion of patients presenting with decompensated liver disease have no prior diagnosis of liver disease despite multiple acute healthcare interactions providing opportunities for detection.

We aimed to characterise patients presenting to unscheduled care with no known liver disease who subsequently had a liver related admission (DLD), and determine if a simple predictive score could identify those at high risk.

**Methods:**

All patients attending unscheduled care in our health board between the beginning of 2018 and the end of 2020 were included with clinical follow up until end 2022. Exclusion criteria were known liver disease, early (< 6 months) presentation with DLD or missing key laboratory data. A predictive model was developed based on demographic and laboratory parameters.

**Results:**

Following exclusions, a group of 173,486 patients were included in our analysis, of whom 1,609 (0.9%) went on to have a DLD-related admission in the 5 year-follow up period. A model to predict future admission was developed based on Fib4 score (using the common blood tests Aspartate aminotransferase (AST), Alanine Transaminase (ALT) and platelet count), geographical deprivation decile, and sex. This model had a Harrell’s C statistic of 0.78.

**Conclusions:**

Unscheduled care presentations provide an opportunity to identify those at high risk of advanced liver disease and decompensation. It is likely these patients have undiagnosed liver disease at the time of presentation, and a model using simple laboratory and demographic data may aid detection in this setting of those at risk of future liver-related admission. External validation of this model is required.

**Supplementary Information:**

The online version contains supplementary material available at 10.1186/s12876-025-04534-2.

## Background

Liver cirrhosis is one of the leading causes of mortality and morbidity in those of working age. Mortality from liver disease in the UK has continued to rise over the past decade compared to improvements in the remainder of Western Europe [1–3]. Unfortunately, a fifth of patients presenting to hospital with decompensated liver disease have no prior diagnosis, and around 15% of those patients will not survive their index admission [4]. For patients with alcohol related cirrhosis, around 50% are diagnosed during a hospital admission rather than in primary or community care [5].

A recent study reported that patients admitted with liver disease due to alcohol had an average of five previous unscheduled care attendances. This suggests missed opportunities to identify those at risk and intervene to prevent future decompensation and liver-related mortality [6]. The recent introduction of opt out viral hepatitis testing in the Emergency Department in NHS England has shown the potential for identifying patients with undiagnosed liver disease through unscheduled care pathways [7].

There is evidence to suggest that early identification of significant liver disease can have an impact on modifiable risk factors such as alcohol use [8]. While there is an increasing awareness of the need to improve detection of liver disease prior to complications arising, many previous studies have focused on primary care identification particularly for those with Metabolic Associated Steatotic Liver Disease [9, 10]. However, those with common liver disease risk factors may not be engaged with community services, with recent data showing a reduction in primary care diagnosis and treatment of alcohol use disorder, in contrast to rising alcohol harm [11]. Furthermore, a meta-analysis of strategies to identify alcohol-related liver disease in those with alcohol use disorder identified the highest yield (between 10.8% and 29.6%) from strategies targeting hospital inpatients rather than community-based programs [12].

Another limitation of current early detection approaches concentrating on risk of liver fibrosis alone, is that the majority of patients identified may never come to harm from their liver disease and, particularly for those with MASLD, have other significant comorbidities [13]. Therefore an alternative approach focusing on patients at risk of liver disease related morbidity, such as a liver related hospital admission, may help concentrate resources on patients most likely to benefit from aggressive interventions.

Among the simple tools available for assessment of liver fibrosis, the FIB4 score is perhaps the most widely studied. This comprises the patient’s age, AST and ALT values, and platelet count. While initially developed for use in viral hepatitis [14], it has also been extensively validated in outpatient populations with metabolic dysfunction-associated steatotic liver disease (MASLD) [15]. While a variety of cut-off values have been proposed, a score < 1.45 points usually excludes significant liver fibrosis, whereas a score > 3.25 points suggests high risk of cirrhosis. Due to alcohol’s impact on platelet count and AST values, it is felt to be less specific for detecting alcohol-related liver fibrosis in the outpatient population [16], but its use in those attending unscheduled care has not been widely explored.

We aimed to use a large regional population and associated data to identify patients without known liver disease presenting to unscheduled care, who subsequently had a liver related hospital admission. We also aimed to evaluate the utility of a model based on FIB4 in addition to demographic variables in predicting future liver related hospital admissions among this patient cohort.

## Methods

A retrospective cohort study was conducted using linked routine collected clinical data. The cohort was identified from hospital episode information for NHS Greater Glasgow and Clyde, the largest health board in Scotland, which provides services to 1.2 million people (approximately a quarter of the Scottish population). All patients attending unscheduled care (Emergency Department and GP referrals to acute admissions) in NHS Greater Glasgow and Clyde Hospitals between 1/1/2018 and 31/12/2020 were identified using the Scottish Morbidity Records, general acute inpatient and day cases and Trak A + E datasets. These patient records were linked to death records provided by National Records of Scotland (NRS) and the NHS Greater Glasgow and Clyde’s Scottish Care Information Store data from the beginning of 2012 to end of 2022, allowing an 8-year lookback and a minimum of 2 years (maximum possible 5 years) electronic patient record data collection after index presentation. The data were pseudonymised and analysed in the West of Scotland Safe Haven, a trusted research environment. Data of interest included prior diagnoses, demographics, laboratory tests and discharge codes for all admissions from 2012 to 2022.

Data extraction and record linkage were performed by the West of Scotland Safe Haven service (IRAS Project ID 321198) at NHS Greater Glasgow and Clyde, under local ethical approval GSH22ME007.

We excluded patients who had a recorded diagnosis of liver disease (defined as the nested International Statistical Classification of Disease and Related Health Problems, 10th Revision (ICD-10) codes K70 or K72, K74, K76.6, K76.7, B16 or B18 in any diagnostic position, see supplementary Table 1) or alcohol-related liver disease (ICD-10 code K70.9 in any position) prior to index unscheduled care episode in the previous available data. We also excluded those with any of these diagnoses recorded during the index admission or any subsequent admission within 180 days of the index unscheduled care attendance to best identify a group where an intervention might change the overall disease outcome.

Development of DLD was defined as having an admission with a liver disease ICD-10 diagnostic code (as detailed above) in any position.

In developing the predictive model, we further excluded patients where a FIB4 score could not be calculated within 24 h of admission date (i.e. no AST, ALT or platelet count available at time of assessment).

The main outcome of interest was a subsequent admission with DLD > 6 months following the index unscheduled care admission. Time to DLD admission was modelled using Cox proportional hazards regression models with censoring occurring at the end of 2022 or at the time of death, whichever occurred earlier. Two models were constructed: (1) with index admission FIB4 fitted as a ordinal variable using low (< 1.45), medium (1.45–3.25) and high risk (> 3.25) categories [14], and (2) with index admission FIB4 fitted using restricted cubic splines (with number of knots determined by likelihood ratio tests, and their values based on Harrell’s recommended percentiles) [17] and age, sex and an area-based measure of socioeconomic deprivation (Scottish Index of Multiple Deprivation, 2016 SIMD deciles - accessed at https://maps.gov.scot/ATOM/shapefiles/SG_SIMD_2016.zip) included as covariates. SIMD uses similar metrics to the Indices of Deprivation (IoD) used in the rest of the UK. The discrimination of the models was estimated using Harrell’s C concordance statistic [18] and by calculating sensitivity, specificity, positive predictive value (PPV) and negative predictive value (NPV) with the risk cut-off determined by those considered high risk by the FIB4 categorical variable approach (for model 2, the value of the regression linear predictor that created a high risk group with the same percentage as model 1 was used).

## Results

Over the study period, 410,422 patients received unscheduled care.

**Fig. 1:**
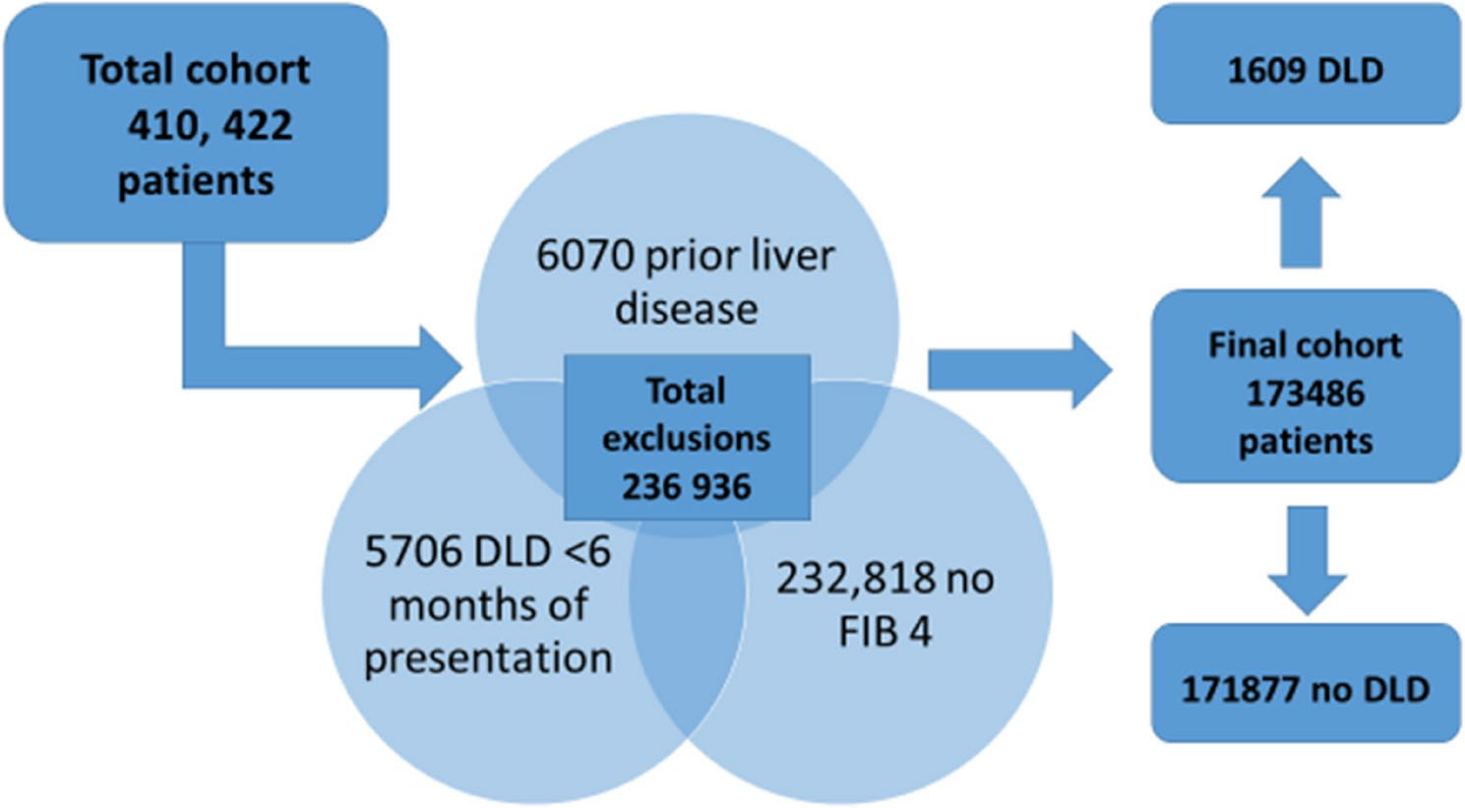
Derivation of final cohort and exclusions

Patients were excluded for prior liver diagnosis (6,070), admission within 180 days with DLD (5706), incomplete laboratory data for FIB 4 (232,818) (See Fig. 1 NB some patients excluded fell into more than one of these categories).

Following exclusions, a total of 173,486 patients were included in our analysis, of whom 1609 (0.9%) went on to have a DLD-related admission within the follow up period. Patients who subsequently developed DLD were more likely to be men and from the highest deprivation deciles (Table 1). Demographics for those excluded due to lack of laboratory data are included in Supplementary Table 2.

Median length of patient record follow-up was 3.6 (inter-quartile range (IQR), 2.6 to 4.4) years. For those patients who developed DLD, the median (IQR) number of unscheduled care admissions following the index admission prior to admission was 7 (4, 12). The overall mortality by the end of the follow up period was 562 deaths (34.9%).

**Table 1:** Cohort characteristic by DLD outcome (number (%) unless indicated)

Variable	Subsequent DLD admission (*n* = 1,609) (% unless otherwise stated)	No subsequent DLD admission (*n* = 171,877) (% unless otherwise stated)	Overall (*n* = 173486) (% unless otherwise stated)
Age, years*	57.0 (15.0)	57.7 (19.8)	57.7 (19.7)
Sex, men	980 (60.9)	78,190 (45.5)	79,170 (45.6)
SIMD deciles			
1 (most deprived)	602 (37.4)	45,273 (26.3)	45,875 (26.4)
2	314 (19.5)	27,052 (15.7)	27,366 (15.7)
3	138 (8.6)	15,949 (9.3)	16,087 (9.3)
4	136 (8.5)	13,690 (8.0)	13,826 (8.0)
5	75 (4.7)	11,567 (6.7)	11,642 (6.7)
6	73 (4.5)	10,136 (5.9)	10,209 (5.9)
7	70 (4.4)	9,310 (5.4)	9,380 (5.4)
8	61 (3.8)	10,671 (6.2)	10,732 (6.2)
9	70 (4.4)	14,157 (8.2)	14,227 (8.2)
10 (least deprived)	49 (3.1)	10,917 (6.4)	10,966 (6.3)
Missing	21 (1.3)	3,155 (1.8)	3,176 (1.8)
FIB4 score**	1.84 (1.09, 3.41)	1.05 (0.66, 1.72	1.06 (0.66, 1.73)
FIB4 categories Low	702 (43.6)	132,719 (77.2)	133,421 (76.9)
Intermediate	477 (30.0)	26,428 (15.4)	26,905 (15.5)
High	430 (26.7)	12,730 (7.4)	13,160 (7.6)

*n* number, *SIMD* Scottish Index of multiple deprivation, *SD* standard deviation.

* mean (SD)

** median (IQR)

The most common ICD-10 discharge codes from unscheduled care for both groups are detailed in Table 2. In initial analyses, some discharge codes, such as epistaxis, appeared to be associated with subsequent DLD (Table 3). However, in generating a model to predict subsequent DLD admission including FIB4 removed the association of DLD with these admission codes.

**Table 2:** Most frequent ICD-10 discharge codes from unscheduled care attendances

Ranking	DLD cohort (*n* = 1,609)	No DLD cohort (*n* = 171,877)
1	Chest pain, unspecified (5.8%)	Chest pain, unspecified (9.5%)
2	Grand Mal Seizures, Unspecified (3.6%)	Other and unspecified abdominal pain (5.5%)
3	Other and unspecified abdominal pain (3.1%)	Other Chest Pain (3.8%)
4	Mental and Behavioural Disorders due to Acute Intoxication with alcohol or drugs (2.8%)	Syncope and Collapse (3.0%)
5	Syncope and collapse (2.6%)	Unspecified Acute Lower Respiratory Infection (2.7%)

**Table 3:** ICD codes associated with subsequent DLD

ICD Code	Description	Percentage primary presentations	HRs (95% CI)
R04	Haemorrhage from respiratory passages (including epistaxis)	0.4%	1.53 (0.79, 2.94)
G40	Epilepsy and recurrent seizures	0.4%	2.26 (1.62, 3.15)
F10	Mental and behavioral disorders due to alcohol use	0.5%	2.66 (2.09, 3.38)

Harrell’s c-statistic was 0.7 for a model using FIB4 alone. A multivariate model was developed, and the simplest model which maintained Harrell’s C statistic was selected. This produced a model incorporating sex, SIMD, and FIB4, which had a Harrell’s C statistic of 0.78.

Within the models generated, FIB4 was significantly associated with subsequent DLD, both as a single predictive variable, and when combined with sex, age and SIMD category (Table 4). Harrell’s C statistics for these models are shown in Table 5. Including the primary discharge code did not substantially improve the proposed model (C statistic 0.7845 with diagnoses included compared with 0.7811 without) therefore these were not included in the final model.

**Table 4:** Cox regression results for time to DLD admission outcome (SIMD decile 1, FIB4 low and female sex used as reference for calculating hazard ratios)

*Model 1 *– FIB 4 alone				
Variable	HR	95% CI		*p*-value
FIB4				
Low	1.00			
Intermediate	3.89	3.47,	4.37	< 0.001
High	9.13	8.10,	10.30	< 0.001
*Model 2 (for FIB4 and age HRs*,* see* Fig. 2*)*				
Sex, male	1.47	1.33,	1.63	< 0.001
Socio-economic deprivation, SIMD				
2	0.91	0.79,	1.04	0.169
3	0.70	0.58,	0.84	< 0.001
4	0.77	0.64,	0.93	0.006
5	0.53	0.42,	0.68	< 0.001
6	0.60	0.47,	0.76	< 0.001
7	0.63	0.49,	0.81	< 0.001
8	0.46	0.35,	0.60	< 0.001
9	0.40	0.32,	0.52	< 0.001
10 (least deprived)	0.36	0.27,	0.48	< 0.001

*SIMD* Scottish Index of Multiple Deprivation, *HR* hazard ratio, *CI* confidence interval

**Table 5:** Predictive accuracy of models: model 1 (not including age) and model 2 (including age)

Follow-up time, years	Sensitivity	Specificity	PPV	NPV
*Model 1 (Harrell’s C concordance = 0.70)*				
1	33.2	94.0	1.1	99.9
2	30.2	94.6	2.8	99.6
3	28.3	95.1	4.7	99.4
4	26.8	95.5	6.4	99.1
5	22.9	95.9	7.8	98.8
*Model 2 (Harrell’s C concordance = 0.78)*				
1	43.6	92.8	1.2	99.9
2	41.6	92.9	3.0	99.7
3	40.0	93.0	4.7	99.5
4	39.1	93.1	6.2	99.3
5	35.5	89.5	4.9	98.9

*PPV* positive predictive value, *NPV* negative predictive value

**Fig. 2:**
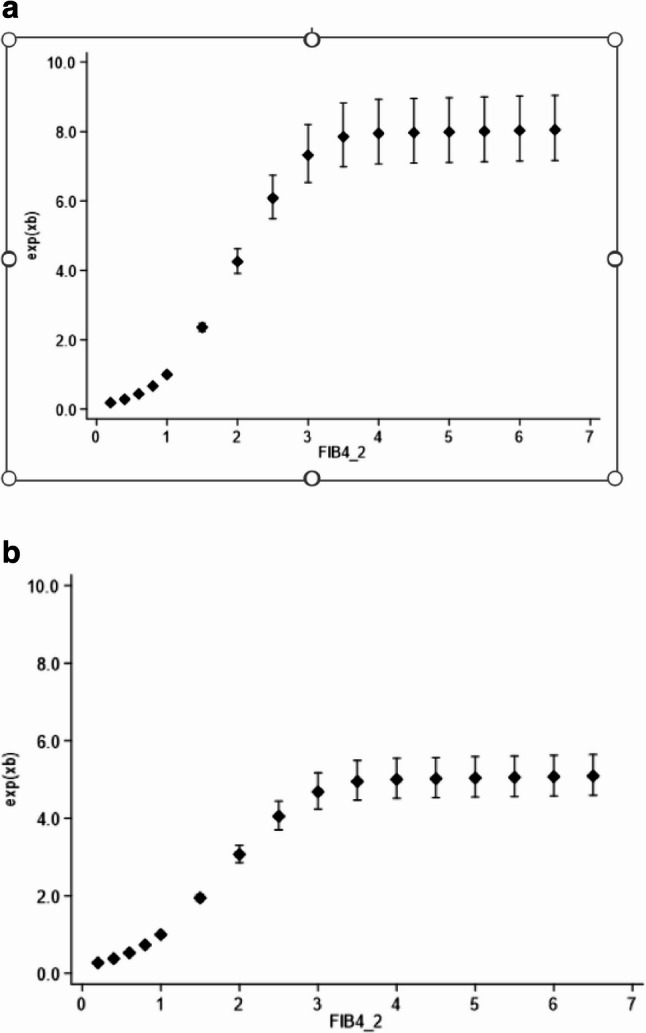
**a **Hazard ratio (exp (xb)) of DLD within the model by FIB4 score if age is included in model 2. **b** Hazard ratio (exp (xb)) of DLD within the model by FIB4 score if age is not included in Model 2

As FIB4 incorporates age, the second model was calculated both including (Fig. 2a) and excluding (Fig. 2b) age as a separate variable in the model. Both models showed increasing hazard ratios (HR) of DLD with increasing FIB4, with the highest HR at FIB4 values above 2.82. In our data set, although age is a component of FIB-4 the correlation between age and FIB-4 is low (correlation coefficient = 0.16) so there is no concern of collinearity in the Cox regression model. As Model 2 (incorporating age) has the strongest prediction, we would propose this as the final model.

This model was generated having excluded those who developed DLD within 180 days of index presentation. This exclusion was in order to ensure our model would identify patients with a window of opportunity for intervention, however any model applied prospectively would identify these patients, therefore we repeated our analysis without this exclusion. 1,561 patients were excluded on this criteria alone. Demographics for these patients can be found in Supplementary Table 3. These patients were more often male, older by a median of 1.6 years and had higher FIB4 scores than the main DLD cohort. If we Include these patients in the final model, the c-statistic improves to 0.806 (from 0.781).

The HR within the model for various FIB4 values is above both including (Fig. 2a) and excluding (Fig. 2b) age. FIB 4 values greater than 2.82 (the top 10% of values) carried a HR of DLD of 8.50 (95% CI 7.48–9.65) in the model including age.

## Discussion

We demonstrated that among included patients presenting to unscheduled care with no prior liver disease diagnosis, almost 1% were admitted with liver disease within 5 years. Furthermore, those who developed DLD had a median 6 further unscheduled care admissions before finally being admitted for DLD, suggesting multiple missed opportunities for intervention. Unsurprisingly, this group had a high mortality rate (34.9% by the end of follow up) despite a median age of 57 years.

The use of unscheduled care rather than primary care for those with alcohol and other substance use disorders means that identification of those at risk of liver disease in this setting may provide a valuable additional opportunity to intervene to prevent subsequent decompensation.

Given that cirrhosis most often develops over decades and the median follow up period of our cohort is under 5 years, those who subsequently present with liver disease already likely have cirrhosis or severe liver fibrosis at the time of their index admission. Therefore, any intervention would take the form of tertiary prevention, aiming to delay and ideally avoid the development of decompensation complications. For many of these patients this is likely to be in the form of intervention for alcohol use disorder for those with alcohol-related liver disease, referral to weight management pathways for MASLD patients and antiviral therapy for those with viral hepatitis.

It is well documented that even in those patients with cirrhosis or severe liver disease, long-term outcome can be influenced by treatment at that point with the opportunity for recompensation. The STOPAH trial in patients with alcohol-related hepatitis showed that in those with severe disease, abstinence from alcohol was the most predictive factor of survival more than 90 days after initial presentation, with other studies demonstrating clear survival advantage in both compensated and decompensated cirrhosis patients achieving abstinence [19, 20].

Scottish figures for Hepatitis C-related hospital admissions also showed a significant reduction following the introduction of effective antiviral therapy, suggesting that decompensation could be averted within a short period if the underlying liver disease aetiology could be treated [21]. Therefore, early identification of advanced liver disease offers an opportunity to alter the natural history of the condition substantially.

The FIB4 score has been widely validated in MASLD for predicting the presence of cirrhosis. Higher values have also been associated with decompensation and liver mortality in the general population [10]. However, the majority of models to predict future liver disease decompensation have been developed in those with known cirrhosis and tend not to include patients with alcohol related liver disease [22, 23]. There are no prior studies examining the utility of FIB4 as a screening tool for the risk of future liver decompensation used in the unscheduled care setting. Previous studies, which have focused on the detection of liver disease during acute admissions, have focused on review by alcohol recovery teams measuring liver stiffness using vibration controlled transient elastography [12]. This approach may be limited by the availability of equipment and trained personnel. It would also miss individuals who present at the Emergency Department but are not admitted or discharged prior to review by the alcohol team. Therefore, the use of a score based on a blood test and demographic information to identify patients at substantial risk of liver disease admission and death has clear benefits over using transient elastography as the initial screening tool this setting.

As with any observational study using secondary analysis of routine data, data points were missing for a proportion of the cohort and we are reliant on the accuracy of diagnostic coding. Due to the anonymised extraction of data we were not able to triangulate admission for liver disease with classical markers of decompensation such as ascites, however the high mortality rate in this group is commensurate with that expected for a population with advanced liver disease.

A primary limitation of this study was that blood tests for FIB4 were frequently not available, leading to exclusion of a substantial number of the cohort. However, our aim was to develop a tool which could be pragmatically applied in the Emergency Department, and it would be unreasonable to expect Emergency Department clinicians to have taken additional blood tests to screen for liver disease if not clinically indicated. Analysis of those excluded due to missing blood tests showed that those who did not get FIB 4 recorded have similar distributions of sex and deprivation to included patients, however this cohort were a mean of 11.8 years younger. This is likely to reflect different index reasons for admission in a younger cohort requiring admission and phlebotomy less often. The excluded cohort had a lower proportion of subsequent DLD (0.6% vs. 0.9%) suggesting that focusing on those with laboratory tests taken selects a group at higher baseline risk. It follows that our findings and developed model can only be applied to those who have appropriate blood tests taken as part of their unscheduled admission.

If validated, this model could be used to automatically flag patients at risk attending the emergency department through the laboratory reporting system. We acknowledge the limitations of the current predictive model as its PPV is relatively low (4.9% for 5 year risk of DLD or 1:20 patients). Currently we would position this as a tool to identify those most at risk for invitation for a further non-invasive liver fibrosis assessment such as transient elastography or reflex biomarker panel testing (e.g. ELF panel [24]).

It should be noted that in targeting prediction of liver related hospital admissions we are focusing on the end of the liver disease spectrum with a high economic and health burden. The cost of decompensation has been estimated at $50,000 per year (£38,200 using HMRC November 2025 exchange rate) whereas the cost of a biomarker panel test is under £200 [25]. We would expect that more than 1:20 patients in the high risk group would have significant liver fibrosis but this requires exploration in further validation studies.

Different modelling methods may be required to allow identification of patients at risk of liver disease without contemporaneous blood tests, including the use of more sophisticated machine learning models to capture non-linear relationships and interaction effects among variables. This is beyond the scope of this project.

Further work is required to externally validate our model and refine the threshold which could be applied in unscheduled care to trigger further investigation and interventions aimed at preventing subsequent decompensation.

## Conclusion

Approximately 0.9% of our cohort, attending unscheduled care with no known liver diagnosis, are subsequently admitted to hospital with liver disease diagnoses. These individuals have high unscheduled care attendance rates with multiple missed opportunities for diagnosis and intervention. Application of a simple model based on the FIB4 score, to patients presenting to unscheduled care who have blood tests taken as part of routine care, predicts subsequent admission for decompensated liver disease. If validated in another cohort, this tool could be routinely administered in Emergency Departments and Acute Receiving Units to identify and signpost patients who would benefit from interventions.

## Supplementary Information

Supplementary Material 1.

## Data Availability

Data analysis was based on the analysis of data held within the West of Scotland Safe Haven. Restrictions apply to the availability of this data, which was used with permission for the current study, so is not publicly available. Datasets used for specific West of Scotland Safe Haven projects may be made available on request, with appropriate ethical permissions and in accordance with standard Safe Haven security policies and procedures. Information on access to Safehaven held data can be found at [West of Scotland Safe Haven - NHSGGC] (https://www.nhsggc.scot/hospitals-services/services-a-to-z/west-of-scotland-safe-haven).

## Abbreviations

ALT: Alanine aminotransferase
ArLD: Alcohol related liver disease
AST: Aspartate aminotransferase
DLD: Decompensated Liver Disease cohort
ED: Emergency Department
MASLD: Metabolic associated liver disease
Non-DLD: Non decompensated liver disease cohort

## References

[1] Public Health Scotland. Scottish Public Health Observatory Update. Edinburgh: PHS; 2025.

[2] Office for Health Improvement and Disparities. Liver Disease Profile 2024: update. London: OHID; 2024. Available from: https://www.gov.uk/government/statistics/liver-disease-profile-april-2024-update/liver-disease-profile-april-2024-update. Cited 2024 June 1.

[3] The Lancet. Tackling liver disease in the UK: a Lancet Commission. Lancet. 2014;384(9958):1902. 10.1016/S0140-6736(14)61820-3. 10.1016/S0140-6736(14)62263-7 25435439

[4] Bodger K, Tapper M, Sterling P, et al. Outcomes of first emergency admissions for alcohol-related liver disease in England over a 10-year period: retrospective observational cohort study using linked electronic databases. BMJ Open. 2023;13(6):e076955. 10.1136/bmjopen-2023-076955. 10.1136/bmjopen-2023-076955 PMC10668174 37993152

[5] Ratib S, Fleming KM, Crooks CJ, et al. One- and five-year survival estimates for people with cirrhosis of the liver in England, 1998–2009: a large population study. J Hepatol. 2014;60(2):282–9. 10.1016/j.jhep.2013.09.027. 10.1016/j.jhep.2013.09.027 24128415

[6] Subhani M, Roberts ER, Baxter JM, et al. Alcohol-related liver disease mortality and missed opportunities in secondary care: a United Kingdom retrospective observational study. Drug Alcohol Rev. 2022;41(6):1331–40. 10.1111/dar.13495. 10.1111/dar.13482 PMC9541852 35640649

[7] Zeng J, Macdonald D, Durkin R, et al. Opt-out testing for hepatitis B and C infections in adults attending the emergency department of a large London teaching hospital. J Clin Virol. 2023;169:105615. 10.1016/j.jcv.2023.105615. 37948983 10.1016/j.jcv.2023.105615

[8] 8. Subhani M, Duffy E, Kerr HJ, et al. Does knowledge of liver fibrosis affect high-risk drinking behaviour (KLIFAD): an open-label pragmatic feasibility randomised controlled trial. EClinicalMedicine. 2023;61:102069. 10.1016/j.eclinm.2023.102069. 10.1016/j.eclinm.2023.102069 PMC10336239 37448808

[9] Abeysekera KWM, Goulston MK, Ortiz-Kemp M, Samuel PR, Roderick HR, Yapp A, et al. Community pathways for the early detection and risk stratification of chronic liver disease: a narrative systematic review. Lancet Gastroenterol Hepatol. 2022;7(8):770–80. 10.1016/S2468-1253(22)00158-1. 10.1016/S2468-1253(22)00020-6 35525248

[10] Åberg F, Van Veldhoven J, Petäjä J. Combined use of the CLivD score and FIB-4 for prediction of liver-related outcomes in the population. Hepatology. 2024;80(1):163–72. 10.1097/HEP.0000000000001524. 38112489 10.1097/HEP.0000000000000707 PMC11191041

[11] Montgomery C, Schofield S, et al. Prevalence and incidence of alcohol dependence: cross-sectional primary care analysis in Liverpool, UK. BMJ Open. 2023;13:e071024. 10.1136/bmjopen-2022-071024. 10.1136/bmjopen-2022-071024 PMC10123861 37076152

[12] Archer AJ, Wang JM, Zhang WZ, Ghosh FH, Marshall MD, Ahmed AA, et al. Proactive case finding of alcohol-related liver disease in high-risk populations: a systematic review. Liver Int. 2024;44(6):1298–308. 10.1111/liv.15812. 38456654 10.1111/liv.15895

[13] Curran C, Priest M, Datta S, et al. Hepatocellular carcinoma risk scores predict patients under surveillance at low risk of benefit and high risk of harm. Dig Dis Sci. 2023;68(3):770–7. 10.1007/s10620-022-07731-1. 36376575 10.1007/s10620-022-07731-1

[14] Sterling RK, Lissen E, Clumeck N, APRICOT Clinical Investigators, et al. Development of a simple noninvasive index to predict significant fibrosis in patients with HIV/HCV coinfection†‡. Hepatology. 2006;43(6):1317–25. 10.1002/hep.21178. 16729309 10.1002/hep.21178

[15] López Tórrez SM, Rodríguez AC, Perez CR, et al. Accuracy of prognostic serological biomarkers in predicting liver fibrosis severity in people with metabolic dysfunction-associated steatotic liver disease: a meta-analysis of over 40,000 participants. Front Nutr. 2024;11:1284509. 10.3389/fnut.2024.1284509. 10.3389/fnut.2024.1284509 PMC10899345 38419854

[16] Moreno C, Mello S, Soriano G. Non-invasive diagnosis and biomarkers in alcohol-related liver disease. J Hepatol. 2019;70(2):273–83. 10.1016/j.jhep.2018.10.015. 10.1016/j.jhep.2018.11.025 30658728

[17] Harrell FE Jr. Regression modeling strategies: with applications to linear models, logistic regression, and survival analysis. New York: Springer; 2001. 10.1007/978-1-4757-3462-1.

[18] Harrell FE, Lee KL, Mark DB. Multivariable prognostic models: issues in developing models, evaluating assumptions and adequacy, and measuring and reducing errors. Stat Med. 1996;15(4):361–87. 10.1002/(SICI)1097-0258(19960229)15:4%3C;361::AID-SIM168%3E;3.0.CO;2-4. 8668867 10.1002/(SICI)1097-0258(19960229)15:4<361::AID-SIM168>3.0.CO;2-4

[19] Lackner C, Spindelboeck W, Haybaeck J, Datz C, Puspok R, Trauner M, et al. Histological parameters and alcohol abstinence determine long-term prognosis in patients with alcoholic liver disease. J Hepatol. 2017;66(3):610–8. 10.1016/j.jhep.2016.11.005. 10.1016/j.jhep.2016.11.011 27894795

[20] Thursz MR, Richardson P, Allison M, et al. Prednisolone or pentoxifylline for alcoholic hepatitis. N Engl J Med. 2015;372(17):1619–28. 10.1056/NEJMoa1412278. 25901427 10.1056/NEJMoa1412278

[21] McDonald SA, Pal K, Bhattacharyya S, et al. Real-world impact following initiation of interferon-free hepatitis C regimens on liver-related outcomes and all-cause mortality among patients with compensated cirrhosis. J Viral Hepat. 2020;27(3):270–80. 10.1111/jvh.13247. 10.1111/jvh.13232 31696575

[22] Gaspar R, Silva M, Lourenço R, et al. Predictive models of mortality and hospital readmission of patients with decompensated liver cirrhosis. Dig Liver Dis. 2019;51(8):1130-7. 10.1016/j.dld.2019.05.017. 10.1016/j.dld.2019.03.016 31113738

[23] Haghnejad V, Burke L, El Ouahabi S, Parker R, Rowe IA. Prediction models for liver decompensation in compensated advanced chronic liver disease: A systematic review. Hepatology. 2025. 10.1097/HEP.0000000000001359. 10.1097/HEP.0000000000001359 40262122

[24] Parkes J, Guha IN, Roderick P, et al. Enhanced liver fibrosis test can predict clinical outcomes in patients with chronic liver disease. Gut. 2010;59(9):1245-51. 10.1136/gut.2009.203166. 10.1136/gut.2009.203166 20675693

[25] Kanwal F, Tapper EB, Ho C, et al. Development and validation of a risk prediction model for cirrhosis decompensation. Am J Gastroenterol. 2024;119(3):497–504. 10.14309/ajg.0000000000002472.

