# Supplementary material for: Identifying patients at high risk of decompensated liver disease through unscheduled care attendance data: a retrospective cohort study
Source: BMC Gastroenterol. 2026 Jan 21;26:126. doi: 10.1186/s12876-025-04534-2 (PMC12905958; doi:10.1186/s12876-025-04534-2)
Supplement: Supplementary file 1 — Supplementary Material 1. [file 12876_2025_4534_MOESM1_ESM.doc]

**Supplementary tables and figures**

**Supplementary Table 1: ICD codes for identification of prior liver disease or liver related admission**

| ICD code | Diagnosis |
| --- | --- |
| K70 | Alcoholic liver disease |
| K72 | Hepatic Failure not elsewhere classified |
| K74 | Fibrosis and Cirrhosis of the liver |
| K76.6 | Portal Hypertension |
| K76.7 | Hepatorenal syndrome |
| B16 | Hepatitis B |
| B18 | Chronic viral hepatitis |
| K70.9 | Alcohol related liver disease |

**Supplementary Table 2: Cohort characteristic by FIB4 recorded status (number (%) unless indicated)**

| Variable | FIB4 recorded (n=173,486) | No FIB4 recorded  (n=232,818) |
| --- | --- | --- |
|  |  |  |
| Age, years* | 57.7 (19.7) | 46.2 (18.4) |
| Sex, men | 79,170 (45.6) | 115,338 (49.5) |
| SIMD deciles |  |  |
| 1 (most deprived) | 45,875 (26.4) | 58,655 (25.2) |
| 2  3  4  5  6  7  8  9  10 (least deprived) | 27,366 (15.8)  16,087 (9.3)  13,826 (8.0)  11,642 (6.7)  10,209 (5.9)  9,380 (5.4)  10,732 (6.2)  14,227 (8.2)  10,966 (6.3) | 34,337 (14.8)  21,090 (9.1)  18,532 (8.0)  16,111 (6.9)  14,435 (6.2)  12,386 (5.3)  16,064 (6.9)  20,092 (8.6)  15,808 (6.8) |
| Missing | 3,176 (1.8) | 5,308 (2.3) |
| Subsequent DLD admission | 1,609 (0.9) | 1,288 (0.6) |

* mean (SD)

  n number; SIMD Scottish Index of multiple deprivation; SD standard deviation

**Supplementary Table 3: Cohort characteristic by DLD outcome – excluding within 180 days vs. only within 180 days (number (%) unless indicated)**

| Variable | Subsequent DLD admission (n=1,609) | Subsequent DLD admission within 180 days  (n=1,561) |  |
| --- | --- | --- | --- |
|  |  |  |  |
| Age, years* | 57.0 (15.0) | 58.6 (14.4) |  |
| Sex, men | 980 (60.9) | 1,000 (64.1) |  |
| SIMD deciles |  |  |  |
| 1 (most deprived) | 602 (37.4) | 562 (36.0) |  |
| 2  3  4  5  6  7  8  9  10 (least deprived) | 314 (19.5)  138 (8.6)  136 (8.5)  75 (4.7)  73 (4.5)  70 (4.4)  61 (3.8)  70 (4.4)  49 (3.1) | 274 (17.6)  141 (9.0)  120 (7.7)  91 (5.8)  80 (5.1)  62 (4.0)  69 (4.4)  91 (5.8)  54 (3.5) |  |
| Missing | 21 (1.3) | 17 (1.1) |  |
| FIB4 score* | 1.84 (1.09, 3.41) | 3.12 (1.43, 6.56) |  |
| FIB4 categories  Low | 702 (43.6) | 468 (30.0) |  |
| Intermediate | 477 (30.0) | 333 (21.3) |  |
| High | 430 (26.7) | 760 (48.7) |  |

* mean (SD) ** median (IQR)
